# Health Department Use of Social Media to Identify Foodborne Illness — Chicago, Illinois, 2013–2014

**Published:** 2014-08-15

**Authors:** Jenine K. Harris, Raed Mansour, Bechara Choucair, Joe Olson, Cory Nissen, Jay Bhatt

**Affiliations:** 1 Brown School, Washington University in St. Louis; 2 Chicago Department of Public Health; 3 FoodBorne Chicago

An estimated 55 million to 105 million persons in the United States experience acute gastroenteritis caused by foodborne illness each year, resulting in costs of $2–$4 billion annually (1). Many persons do not seek treatment, resulting in underreporting of the actual number of cases and cost of the illnesses (2). To prevent foodborne illness, local health departments nationwide license and inspect restaurants (3) and track and respond to foodborne illness complaints. New technology might allow health departments to engage with the public to improve foodborne illness surveillance (4). For example, the New York City Department of Health and Mental Hygiene examined restaurant reviews from an online review website to identify foodborne illness complaints (5). On March 23, 2013, the Chicago Department of Public Health (CDPH) and its civic partners launched FoodBorne Chicago (6), a website ( https://www.foodbornechicago.org ) aimed at improving food safety in Chicago by identifying and responding to complaints on Twitter about possible foodborne illnesses. In 10 months, project staff members responded to 270 Twitter messages (tweets) and provided links to the FoodBorne Chicago complaint form. A total of 193 complaints of possible foodborne illness were submitted through FoodBorne Chicago, and 133 restaurants in the city were inspected. Inspection reports indicated 21 (15.8%) restaurants failed inspection, and 33 (24.8%) passed with conditions indicating critical or serious violations. Eight tweets and 19 complaint forms to FoodBorne Chicago described seeking medical treatment. Collaboration between public health professionals and the public via social media might improve foodborne illness surveillance and response. CDPH is working to disseminate FoodBorne Chicago via freely available open source software

FoodBorne Chicago tracked Twitter messages using a supervised learning algorithm (7). The algorithm parsed tweets originating from Chicago that included “food poisoning” to identify specific instances of persons with complaints of foodborne illness. The geographic boundaries used by the algorithm also included some neighboring Chicago suburbs. However, follow-up inspections were conducted only at restaurant locations within the city limits. Tweets identified by the algorithm were reviewed by project staff members for indications of foodborne illness (e.g., stomach cramps, diarrhea, or vomiting) from food prepared outside the home. Project staff members provided feedback on whether each tweet fit the criteria, enabling the tweet identification algorithm to learn and become more effective over time.

For tweets meeting the criteria, project staff members used Twitter to reply. For example, *Tweet:* “Guess who’s got food poisoning? This girl!” *Reply:* “That doesn’t sound good. Help us prevent this and report where you ate here (link to Foodborne Chicago and a web form to report the illness).” The information in submitted forms went directly into the Chicago 311 system that handles all requests for nonemergency city services. Descriptive statistics were used to evaluate FoodBorne Chicago over its first 10 months of use and to compare the results of complaint-based health inspections of food establishments resulting from FoodBorne Chicago use with health inspections of food establishments based on complaints not submitted through FoodBorne Chicago. The comparisons did not include reinspections or routine inspections not based on a complaint.

During March 2013–January 2014, FoodBorne Chicago identified 2,241 “food poisoning” tweets originating from Chicago and neighboring suburbs. From these, project staff members identified 270 tweets describing specific instances of persons with complaints of foodborne illness. Eight of the 270 tweets (3.0%) mentioned a visit to a doctor or an emergency department. A total of 193 complaints of food poisoning were submitted through the FoodBorne Chicago web form. However, project staff members were not able to track how many of the 193 came from persons led to the form via Twitter and how many came from persons who visited the FoodBorne Chicago site on their own.

Of the 193 FoodBorne Chicago complaints, 19 (9.8%) persons indicated they sought medical care. The complaints identified 179 Chicago restaurant locations; at 133 (74.3%) locations, CDPH inspectors conducted unannounced health inspections. These 133 inspections amounted to 6.9% of the 1,941 health inspections of food establishments prompted by complaints during the study period. Of the 133 FoodBorne Chicago–prompted health inspections, 122 (91.7%) inspection reports identified at least one health violation, compared with 91.8% of inspection reports following complaints filed outside of FoodBorne Chicago during the same period.

Of the 133 FoodBorne Chicago–prompted health inspections 27 (20.3%) identified at least one critical violation, compared with 16.4% of the 1,808 inspections not prompted by FoodBorne Chicago. Critical violations indicate an “immediate health hazard” resulting in a high risk for foodborne illness. Critical violations must be fixed while the inspector is present or the restaurant fails inspection, has its license suspended, and is closed.* Twenty-nine restaurants (21.8%) reported via FoodBorne Chicago had at least one serious violation compared with 27.8% of restaurants not reported via FoodBorne Chicago. Serious violations indicate a “potential health hazard” that must be corrected within a timeframe determined by the health inspector, typically 5 days. If the serious violation is not fixed on re-inspection, the license is suspended, and the business is closed. Overall, at least one critical or serious violation was found in 37.6% of inspections prompted by FoodBorne Chicago and 37.2% of inspections from other complaints during the same period.

Some differences were noted in the distribution of specific violations between FoodBorne Chicago inspections and other complaint inspections. For example, 13.5% of FoodBorne Chicago inspections resulted in (critical) violation 3 (i.e., food not stored at appropriate temperatures), compared with 8.2% of other complaint inspections (Table). In addition, 14.3% of other complaint inspections reported (serious) violation 18 (i.e., food not protected from contamination), compared with 6% of FoodBorne inspections.

A total of 21 (15.8%) of the 133 restaurants reported through FoodBorne Chicago failed inspection and were closed; an additional 33 restaurants (24.8%) passed with conditions, indicating that serious or critical violations were identified and corrected during inspection or within a specified timeframe. Of the inspected restaurants with complaints not reported through FoodBorne Chicago, 25.8% failed and 14.2% passed with conditions. During the study period, among all restaurants inspected, FoodBorne Chicago–prompted inspections accounted for 4.3% of failed inspections and 11.4% of pass with conditions inspections.

## Discussion

Foodborne illness is a serious and underreported public health problem with high health and financial costs. Emerging evidence on the effectiveness of social media for foodborne illness surveillance suggests mining tweets and restaurant reviews might aid in identifying and taking action on localized foodborne illness complaints that would otherwise go unreported (5,8,9). Using a new surveillance and response strategy, the CDPH identified and responded to 270 tweets about foodborne illness over 10 months in the Chicago area; 193 Chicago FoodBorne forms reporting foodborne illness were filed during this period. The majority of the 193 forms did not indicate that medical treatment was sought and so would likely not have been included in the usual surveillance numbers nor prompted inspections by the health department. Twenty-one of the reported restaurants failed inspection and were closed; 33 additional restaurants passed with conditions. Rates of critical and serious violations and failing inspections prompted by FoodBorne Chicago complaints were similar to those from inspections in response to other complaints during the same period.

The findings in this report are subject to at least two limitations. First, the Twitter application programming interface does not allow precise geographic filtering, and FoodBorne Chicago only used the keyword “food poisoning” to identify tweets. Second, it was not possible to determine how many of the 193 web form complaints were from persons directed to the form via Twitter. Project staff members were able to link 30 tweets directly to a corresponding complaint when report submitters clicked on the link in the “reply tweet” to access and complete the form. However, the number of persons who tweeted, did not click the link, and later accessed the Foodborne Chicago web form is unknown.

CDPH food inspectors and supervisors initially were concerned that use of Twitter would overburden them with increased inspections. However, by understanding the process better and seeing the success in finding violations, CDPH staff members have become supportive of obtaining potential foodborne illness information via Twitter.

CDPH and its partners are actively working to improve and disseminate the FoodBorne Chicago program. In an effort to increase the effectiveness of staff replies to complaints via Twitter, CDPH held four focus groups and plans an online survey. In addition, CDPH is currently working with the Boston Public Health Commission and the New York City Department of Health and Mental Hygiene to adapt FoodBorne Chicago for use in those two cities. FoodBorne Chicago also is available as open-source software on GitHub, an online host for sharing computer code with the public or a private audience.†

What is already known on this topic?

Foodborne illness is a serious and underreported public health problem with high health and financial costs. Local health departments nationwide license and inspect restaurants to prevent foodborne illness and track and respond to foodborne illness complaints. Emerging evidence on the effectiveness of social media for foodborne illness surveillance suggests mining tweets and restaurant reviews might aid in identifying and taking timely action on sources of foodborne illness that would otherwise go unreported.

What is added by this report?

A new open-source surveillance and response tool was used to identify and respond to tweets about foodborne illness in Chicago. Over a 10-month period, the tool identified 133 Chicago-area restaurants that were subsequently inspected. Of these, 21 (15.8%) failed inspection, and 33 (24.8%) passed with conditions.

What are the implications for public health practice?

New technology applied to widely used social media platforms might allow health departments to engage the public to improve foodborne illness surveillance.

## Figures and Tables

**TABLE:** Number and percentage of complaints reported via FoodBorne Chicago and from other sources with subsequent Chicago Department of Public Health (CDPH) inspections, by violation type — Chicago, Illinois, March 2013–January 2014

CDPH violation no.	Health standard	Complaints via FoodBorne Chicago	Complaints from other sources
	
		No. (%)	No. (%)
**Critical violations**			
V1	All food shall be from sources approved by health authorities and safe for human consumption. Shellfish shall be obtained from an approved source and kept in their original package until sold. Molluscan shell stock shall be obtained in containers bearing legible source identification tags or labels.	2 (1.5)	17 (0.9)
V2	All food establishments that prepare, sell, or store hot food shall have adequate hot food storage facilities. All food establishments that display, prepare, or store potentially hazardous food shall have adequate refrigerated food storage facilities.	10 (7.5)	77 (4.3)
V3	All hot food shall be stored at a temperature of 140°F (60°C) or higher. All cold food shall be stored at a temperature of 40°F (4°C) or less.	18 (13.5)	148 (8.2)
V4	All food shall be protected from contamination and the elements, and so shall all food equipment, containers, utensils, food contact surfaces and devices, and vehicles.	3 (2.3)	3 (0.2)
V5	No person affected with or carrying any disease in a communicable form or afflicted with boils, infected wounds, sores, acute respiratory infection, or intestinal disorder shall work in any area of a food establishment in any capacity where there is a likelihood of that person contaminating food or food contact surfaces.	1 (0.8)	0 —
V6	All employees who handle food shall wash their hands as often as necessary to maintain a high degree of personal cleanliness and should conform to hygienic practices prescribed by the Board of Health.	2 (1.5)	24 (1.3)
V7	Hand washing of all tableware and drinking utensils shall be accomplished by the use of warm water at a temperature of 110°F (43°C) to 120°F (49°C) containing an adequate amount of detergent effective to remove grease and solids.	0 —	1 (0.1)
V8	Equipment and utensils should get proper exposure to the sanitizing solution during the rinse cycle. Bactericidal treatment shall consist of exposure of all dish and utensil surfaces to a rinse of clean water at a temperature of not less than 180°F (82°C).	3 (2.3)	24 (1.3)
V9	All food establishments shall be provided with an adequate supply of hot and cold water under pressure properly connected to the city water supply.	0 —	28 (1.5)
V10	In food establishments, there shall be adequate sewage and waste water disposal facilities that comply with all requirements of the plumbing section of the Municipal Code of Chicago.	0 —	8 (0.4)
V11	Adequate and convenient toilet facilities shall be provided. They should be properly designed, maintained, and accessible to employees at all times.	1 (0.8)	30 (1.7)
V12	Adequate and convenient hand washing facilities shall be provided for all employees.	1 (0.8)	36 (2.0)
V13	All necessary control measure shall be used to effectively minimize or eliminate the presence of rodents, roaches, and other vermin/insect infestations.	0 —	8 (0.4)
V14	A separate and distinct offense shall be deemed to have been committed for each serious violation that is not corrected upon re-inspection by the health authority.	0 —	3 (0.2)
**Serious violations**			
V15	Food once served to a consumer shall not be re-served, with the exception of packaged food remaining in its original, unopened package.	0 —	0 —
V16	All food should be properly protected from contamination during storage, preparation, display, service, and transportation.	3 (2.3)	46 (2.5)
V17	Thawing frozen food for further processing shall be accomplished by storage in a refrigerator at 40°F (4°C) or less, or by other approved method.	0 —	0 —
V18	All necessary control measures shall be used to effectively minimize or eliminate the presence of rodents, roaches, and other vermin and insects on the premises of all food establishments, in food-transporting vehicles, and in vending machines.	8 (6.0)	259 (14.3)
V19	The area outside of the establishment used for the storage of garbage shall be clean at all times and shall not constitute a nuisance.	3 (2.3)	46 (2.5)
V20	All garbage and rubbish containing food wastes shall, prior to disposal, be stored in metal containers with tight fitting lids and shall be kept covered except when opened for the disposal or removal of garbage.	0 —	0 —
V21	A certified food service manager must be present in all establishments at which potentially hazardous food is prepared or served.	10 (7.5)	135 (7.5)
V22	All dishwashing machines shall maintain proper water pressure and must be provided with suitable thermometers, chemical test kits, and gauge cocks.	0 —	1 (0.1)
V23	Dishes and other utensils shall be rinsed or scraped to remove gross food particles and other soil before washing.	0 —	0 —
V24	All dishwashing machines must be of a type that complies with all requirements of the plumbing section of the Municipal Code of Chicago and Rules and Regulation of the Board of Health	3 (2.3)	30 (1.7)
V25	Only such poisonous and toxic materials as are required to maintain sanitary conditions may be used in food establishments and they shall not be used in any hazardous manner.	0 —	2 (0.1)
V26	When toilet and lavatory facilities are provided for the patrons of food establishments, such facilities shall be adequate in number, convenient, accessible, properly designed, and installed according to the municipal code.	0 —	20 (1.1)
V27	In all food establishments, toilet facilities shall be kept clean and in good repair and shall include an adequate supply of hot and cold or tempered water, soap, and approved sanitary towels or other approved hand-drying devices.	0 —	1 (0.1)
V28	One copy of the Food Inspection Report Summary must be displayed and visible to all customers.	3 (2.3)	14 (0.8)
V29	A separate and distinct offense shall be deemed to have been committed for each minor violation that is not corrected upon reinspection by the health authority.	5 (3.8)	67 (3.7)

## Footnotes

* Additional information regarding critical and serious violations is available at http://www.cityofchicago.org/city/en/depts/cdph/provdrs/environ_health/svcs/understand_healthcoderequirementsforfoodestablishments.html.

† Additional information is available at https://github.com/smartchicago/foodborne.
